# Gibberellin induced shot berry formation in cv. Early Sweet is a direct consequence of high fruit set

**DOI:** 10.1038/s41438-020-00388-9

**Published:** 2020-10-01

**Authors:** Etti Or, Orly Oren, Tamar Halaly-Basha, Padmalatha Koilkonda, Zhaowan Shi, Chuanlin Zheng, Atiako Kwame Acheampong

**Affiliations:** 1 Institute of Plant Sciences, Department of Fruit Tree Sciences, Agricultural Research Organization, Volcani Center, Rishon LeZion, 7528809 Israel; 2 Division of Crop Sciences, ICAR-Central Research Institute for Dryland Agriculture, Santoshnagar, Hyderabad, Telangana 500059 India; 3 Department of Fruit Tree Sciences, College of Horticulture, China Agricultural University, Beijing, 100193 China; 4 Biology department, University of North Carolina, Chapel Hill, NC 27514 USA

**Keywords:** Plant morphogenesis, Developmental biology

## Abstract

The ‘seedless’ table grape industry relies mainly on stenospermocarpic cultivars, in which endosperm abortion results in berries with seed rudiments and low levels of bioactive gibberellin (GA). Application of GA to enhance berry sizing in these cultivars is often accompanied by adverse effects, one of which is increased proportions of very small berries (termed shot berries). Manual removal of these berries, which is essential to improve uniformity and market value, increases production cost and exposes the cluster to damage. Unraveling the physiological causes of shot berry formation is thus of both scientific and practical value. This study focuses on understanding the GA-mediated regulation of shot berry formation in *Vitis vinifera* cv. Early Sweet, known for a high proportion of shot berries, which severely damage cluster appearance. As GA is known to induce the parthenocarpic fruit set, we first tested the assumption that the parthenocarpic nature of a fruitlet is a primary cause for shot berry development. We then examined the consequence of the flower load on the proportion of shot berries in the cluster. Our data suggests that: (1) contrary to prior assumptions, the parthenocarpic nature of a fruitlet is not the primary cause for shot berry development, demonstrated by the fact that parthenocarpic fruitlets develop into a full-size berries; (2) the proportion of shot berries on a cluster is a function of the initial flower load on the inflorescence, with high initial flower load resulting in greater shot berry percentage in the cluster; (3) GA treatment bypasses the natural regulation of flower load, resulting in high fruitlet density and increased competition among fruitlets; (4) variation of flower load within the cluster influences berry size uniformity to a greater extent than does the variation in number of cluster per vine. The identity of the factors that determine the fate of a given flower on a high-load cluster remains an open question.

## Introduction

The presence of an excessive number of small berries in a cluster with full-sized berries, known as *Millerangdage*^1^, resulting in poor cluster appearance that is a major drawback in the marketing of premium table grape. Manual removal of the small-sized berries (termed shot berries) is required to improve the uniformity of such clusters. Such treatment significantly increases production cost and exposes the cluster to mechanical damage. Despite its importance, knowledge about the factors underlying the formation of shot berries is limited^1^. It was reported that the proportion of shot berries increased in vines with high vegetative vigor^2^, and decreased in response to shoot topping^3^ and when vines are pruned late^4,5^. Different sizes were assigned to shot berries in the literature, from 2 to 9 mm, depending on the cultivar and the variability in berry size within a cluster^1,6,7^. Studies also show that the histological structure of shot berries at harvest was similar to that of a young normal berry^6^, and data suggested that they are characterized by lower sugar and amino acid content^8^. Formation of shot berries has been attributed to defects in fertilization, leading to parthenocarpic fruit set and failure to develop into normal berries^1^. It was reported that the septum of shot berries is lignified, which may testify that fertilization is inhibited, resulting in parthenocarpic berries^6^. Accordingly, the correlation between shot berry formation and environmental conditions that prevents fertilization was proposed^1,6,9^.

Parthenocarpy arises when the fruit develops in the absence of fertilization. Both external applications and molecular studies confirmed that changes in the synthesis and metabolism of auxin and GA, or in components within their signaling pathways, may induce parthenocarpy. Interestingly, GA appears to be the last step in that hormonal cascade^10,11^. In agreement, the pre-anthesis application of GA is used for true seedless berry production using various seeded varieties^12^. However, natural parthenocarpy is rare in *Vitis vinifera*^1^. Strengthening the assumption that parthenocarpy causes small berry development, is the fact that the known parthenocarpic cultivar, cv. Current, has very small berries that resemble shot berries. Nevertheless, many stenospermocarpic grapevine varieties are also characterized by small berry size. Stenospermocarpy, common in *Vitis vinifera*, is caused by endosperm abortion following fertilization, leading to the cessation of seed development, and results in variable size of seed rudiments, and low bioactive GA quantities^1,13,14^. Thus, GA application after fruit set is routinely used for berry sizing in these cultivars that are the backbone of the ‘Seedless’ grape industry^1,15–18^.

It was suggested that environmental, hormonal or nutritional factors that interfere with fertilization might lead to parthenocarpic fruit set rather than to flower abscission, in both stenospermocarpic and seeded grapevine varieties^1,19,20^. This raises the question of whether the formation of shot berries on stenospermocarpic clusters is a result of induced production of parthenocarpic fruitlets, which do not respond to GA sizing treatment, and thus cannot develop to normal size berries like their stenospermocarpic neighbors.

In addition to natural factors that may induce partial parthenocarpy in grapevine clusters, horticultural practices may induce it as well. In agreement, GA applied for thinning and/or for sizing may lead to parthenocarpy if applied too early, when part of the flowers are at pre-anthesis stage. In agreement with the potential connection between parthenocarpy and shot berry formation, GA appears to have an adverse effect on shot berry intensity in stenospermocarpic cultivars, depending on the timing of application^1,21^. For example, (1) GA_3_ application in cv. Perlette before anthesis increased shot berry intensity but did not affect pollen germination^22^; (2) application of GA_3_ during the flowering of cv. Crimson resulted in an increased proportion of shot berries^23^; (3) application of GA to both seeded and seedless varieties at pre-anthesis led to a high incidence of shot berry formation^24^. This study focuses on understanding the adverse effect of GA application on shot berry production in stenospermocarpic cultivars. Cv. Early Sweet, an early ripening variety which is widely grown in warm regions, is very susceptible to shot berry formation, and thus provides a prism through which to dissect the interaction between GA and shot berry formation. In this study, we show that a high proportion of shot berry formation in cv. Early Sweet is a direct consequence of the high fruit set, while parthenocarpy per se will not induce shot berry formation. The results also suggest that (1) reduction of flower number in the inflorescence may limit shot berry percentage and improve size uniformity; (2) the influence of variations in flower load within the cluster is greater than variation in the number of cluster per vine.

## Results

**Fig. 1:**
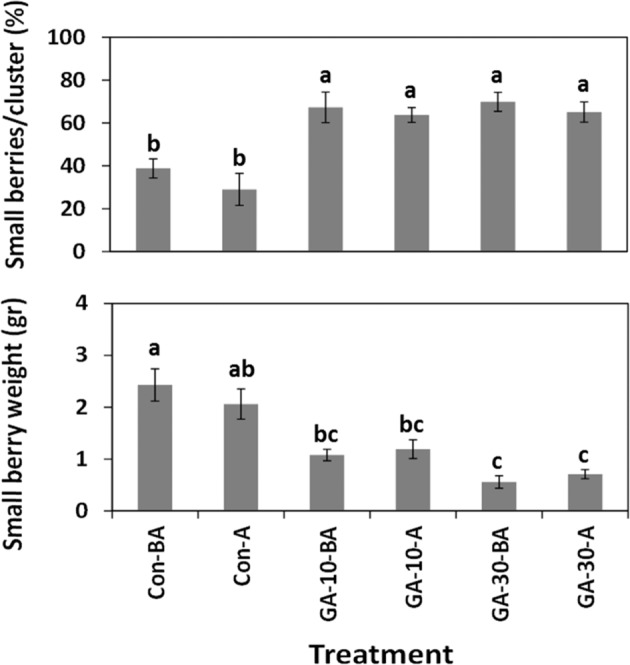
The effect of GA application on shot berry intensity in *Vitis vinifera* cv. Early Sweet. Three groups of 10 inflorescences were labeled and thinned to carry 5 top branches without adjustment of the number of flowers. Two groups were dipped in GA_3_ (10 ppm, 30 ppm), and one group was dipped in water and used as control. All the solutions contained Triton X-100 (0.025%) as the surfactant. The experiment was carried out at two time points: 2 weeks before anthesis (BA) and at anthesis (A). Clusters were collected at harvest, and data were documented for each individual cluster. **a** The percentage of small berries was calculated in relation to the total number of berries. **b** Small berry weight was calculated by dividing the total weight of all small berries by their number. Values are averages of the values in 10 clusters in each treatment ± SE. Different letters between columns indicate significantly different values (*p* < 0.05, Tukey HSD).

We analyzed the ability of GA_3_ to induce variations in the size of berries of cv. Early Sweet. Compared with the control treatments, application of GA_3_, at anthesis or 2 weeks earlier, led to a significant increase in the percentage of small berries in a cluster, based on a visual categorization of all the berries into “big” and “small” berry pools (Fig. 1a). The percentage of small berries was similar in both GA concentration used. GA_3_ reduced the weight of berries in the “small” berry pool in a concentration-dependent manner (Fig. 1b). Additional data related to berry number and weight in the different size categories were recorded (Fig. S1), and will be considered in the discussion.

**Fig. 2:**
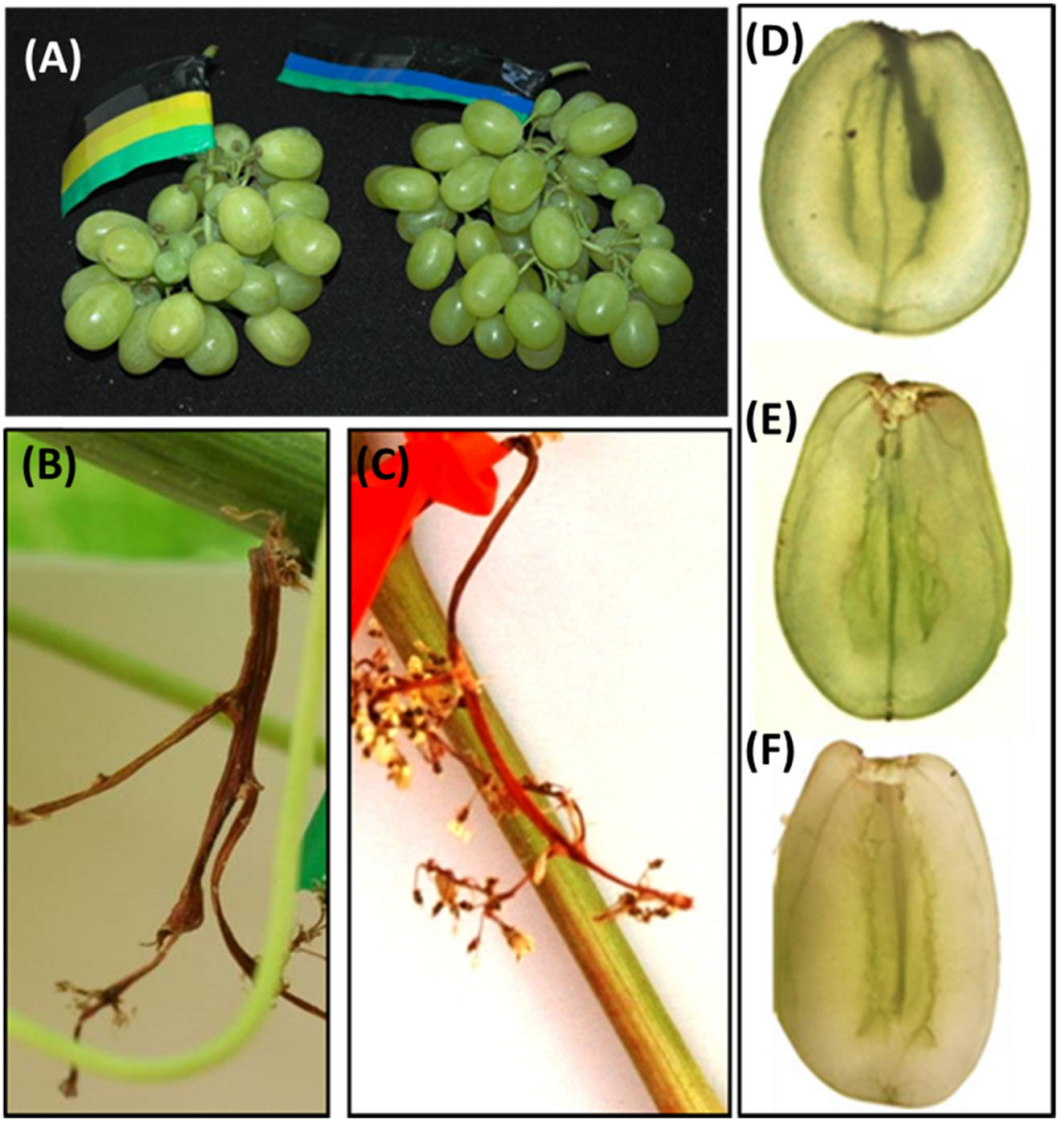
GA application induces a parthenocarpic fruit set of emasculated cv. Early Sweet flowers. In parallel with the experiment described in Fig. 1, five additional groups of clusters were thinned to carry 5 top branches. Here, all 5 branches were thinned to carry 10 flowers each, creating inflorescences with 50 flowers. In 2 of these 5 groups, flowers were emasculated by manual removal of calyptra and anthers. Pairs of emasculated and non-emasculated groups were dipped in GA_3_ (10 ppm), and water. The remaining group of non-emasculated 50 flowers containing inflorescences was treated with higher concentration of GA_3_ (30 ppm). An additional group of clusters, in which flower number was not manipulated, was treated with Paclobutrazol (PAC; 250 ppm). **a** Emasculated (left) and non-emasculated (right) clusters treated with GA_3_ (10 ppm). **b** Emasculated cluster treated with water. **c** Non-emasculated cluster treated with PAC. **d**–**f** Demonstrate a representative berry from non-emasculated cluster treated with water, non-emasculated cluster treated with GA_3_ (10 ppm), and emasculated cluster treated with GA_3_ (10 ppm), accordingly. All the other details are as described in Fig. 1.

**Fig. 3:**
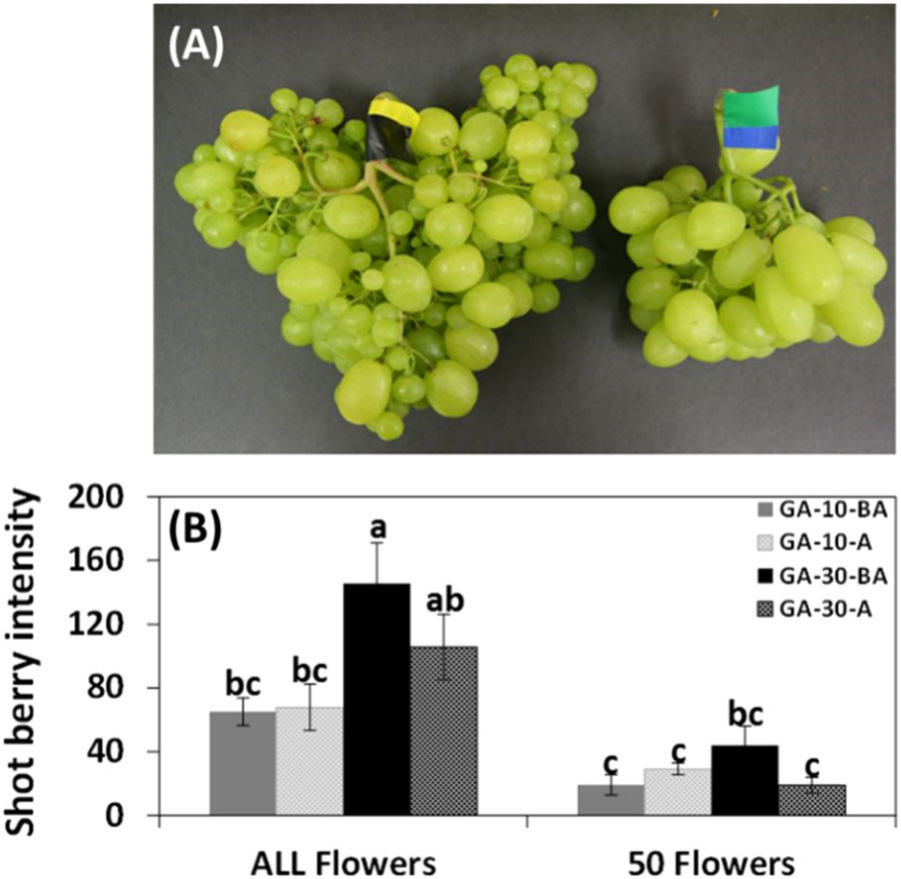
The effect of decreased flower load on shot berry intensity in GA-treated inflorescences. Four groups of 10 inflorescences were thinned to carry 5 top branches. In 2 out of 4 groups, all 5 branches were thinned to carry 10 flowers each, creating inflorescences with 50 flowers. Pairs of groups (“ALL” and “50”) were dipped in GA_3_ (10 ppm, 30 ppm). **a** Representative GA_3_ (10 ppm)-treated clusters with manipulated (right) and non-manipulated (left) number of flowers. **b** Shot berry intensity values calculated for all the treated groups by multiplication of average of small berry weight by the average percentage of small berries in a cluster. All the other details are as described in Figs. 1 and 2.

To test if GA will induce parthenocarpy in cv. Early Sweet, and if parthenocarpic berry will develop into shot berry or into a full-size berry, we modified groups of inflorescences to carry 50 flowers, emasculated the flowers in a sub-group, and treated emasculated and non-emasculated inflorescence with GA_3_ (10 ppm). The results suggest that: (1) GA induce parthenocarpic fruit set in cv. Early Sweet when applied 2 weeks before anthesis (Fig. 2a); (2) GA is indispensable for inflorescence survival, as untreated emasculated inflorescences, as well as PAC-treated non-emasculated inflorescences wilted and dried a few days after treatment (Fig. 2b, c); (3) GA treatment of emasculated flowers resulted in normal-sized, parthenocarpic berries (Fig. 2a, d–f).

To further understand the effect of GA application on size variability, we compared the response of inflorescences manipulated to carry 50 flowers and non-manipulated inflorescences to GA. This comparison revealed a significant decrease in size variability in the manipulated inflorescences when compared with the non-manipulated inflorescences (Fig. 3a). To properly evaluate the effect of GA on size uniformity within the cluster, we used both percentages of small berries within a cluster and the average size of a small berry to describe shot berry intensity (average small berry percentage × average small berry weight; Fig. 3b), in manipulated and non-manipulated inflorescence. It appeared that (1) increasing the GA concentration increased the shot berry intensity in the cluster; (2) decrease in initial load resulted in a decrease in shot berry intensity. These results indicated that while GA induces parthenocarpic fruit set, parthenocarpy itself is not the cause for size variability. Rather, it seems that the combination of high flower load and GA treatment is responsible for the enhanced size variability.

**Fig. 4:**
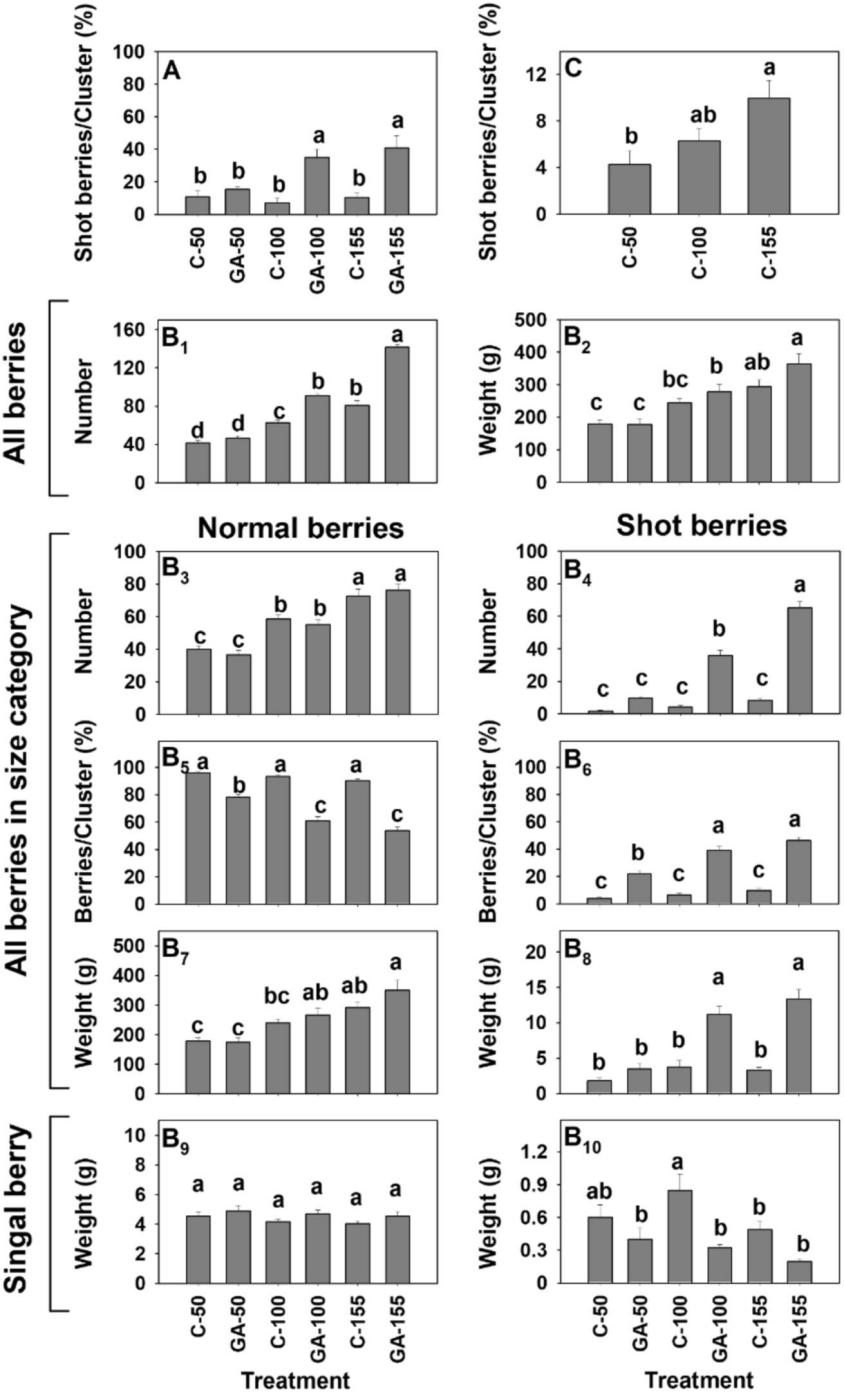
The effect of gradual manipulation of initial flower load on shot berry percentage. Six groups of inflorescences (5 inflorescences per group in the first season and 25 in the following season) were thinned to carry 5 top branches at 2 weeks before anthesis. Pairs of groups were designed for a load of 155, 100, or 50 flowers per inflorescence. For a load of 155 flowers, 40 flowers were left on each shoulder, and 25 flowers were left on the 3 branches below. For a load of 100 and 50 flowers, 20 and 10 flowers were left on each branch, respectively. Of the two groups set for each load, one was treated with GA_3_ (30 ppm). The other group was treated with water and used as control. **a** Percentage of shot berry per cluster for GA-treated and control inflorescences with 50, 100, and 155 flowers, in the experiment that was performed during the first season. **b**_1_–**b**_10_ Numbers and weights of all berries per cluster, berries per size category, and weight of single berry per size category in the experiment that was performed during the following season. **c** Percentage of shot berry per cluster for control inflorescences with 50, 100, and 155 flowers. Here, data from the experiment described in (**b**) was used for a separate statistical analysis of control treatments. All the other details are as described in Fig. 1 and in “Methods”.

To better evaluate the effect of flower load on the degree of shot berry development, inflorescences manipulated to carry 50, 100, and 155 flowers were treated with GA_3_ (30 ppm). Our data revealed that increased flower load resulted in an increased percentage of shot berries within GA-treated clusters (Fig. 4a). Clusters carrying 100 and 155 berries had twice as many shot berries as clusters carrying 50 berries. However, there was no significant difference in shot berry formation for clusters manipulated to carry 100 and 150 flowers. GA treatment did not significantly influence shot berry formation in clusters carrying 50 flowers.

To further study the effect of flower load and GA treatment, identical experiment of a larger scale was conducted (Fig. 4b). In agreement with the above, clusters carrying 100 and 155 berries presented considerable increase in percentage of shot berries within GA-treated clusters, while a much milder difference was recorded for clusters carrying 50 berries (Fig. 4b_6_). Detailed cluster analysis also revealed that (1) there is a significant increase in total number of berries (Fig. 4b_1_), when GA-treated clusters that carry 100 and 150 flowers are compared to the relevant control, but it is not accompanied by significant difference in the total berry weight (Fig. 4b_2_), although such a trend is visible; (2) GA treatment has no significant effect on the number of normal berries (Fig. 4b_3_). It also did not have a significant effect on the weight of the normal berry fraction (Fig. 4b_7_), or the average weight of a single normal berry (Fig. 4b_9_); (3) GA treatment resulted in significant increase in the number of shot berries in clusters manipulated to carry 100 and 150 flowers (Fig. 4b_4_). It also led to increase in shot berry fraction weight (Fig. 4b_8_), and a decrease in the weight of a single shot berry, which was significant only in clusters manipulated to carry 100 berries (Fig. 4b_10_). Similar behavior was observed for the experiment described in Fig. 4a (data are not shown).

A separate analysis of the percentage of small berries in control clusters with different flower loads (Fig. 4c) suggested that higher initial load led to a significantly higher percentage of shot berries in untreated clusters as well.

**Fig. 5:**
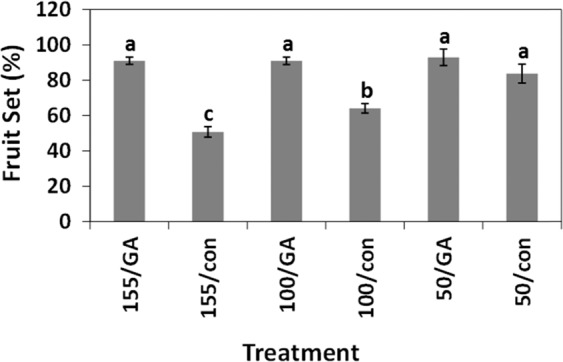
The effect of initial flower load and GA on fruit set percentage in cv. Early Sweet clusters. Data from the experiment described in Fig. 4b was used to calculate the percentage of fruit set with reference to the initial number of flowers. All the other details are as described in Figs. 1 and 4.

To calculate the percentage of fruit set, we compared the initial number of flowers on a cluster to its final number of berries. Irrespective of flower load, GA treatment resulted in a 90% fruit set (Fig. 5). The percentage of fruit set within control inflorescences was significantly lower than that of the GA-treated inflorescences with 100 and 150 flowers, but was not significantly different for a cluster with an initial load of 50 flowers. These results suggest that: (1) higher initial flower load resulted in lower percent of fruit set; (2) there is an inherent mechanism that regulates flower load and ensures optimal cluster size; (3) this mechanism may be disrupted by GA.

**Fig. 6:**
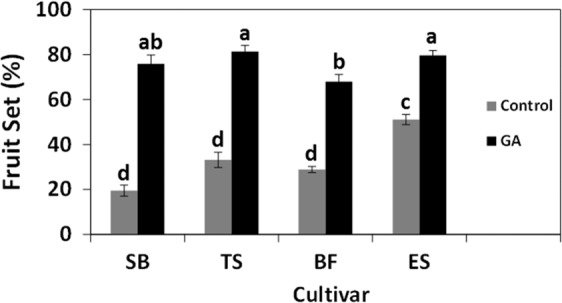
Comparative analysis of the effect of GA on fruit set percentage of several *Vitis vinifera* cultivars. Two groups of 25 inflorescences were thinned 2 weeks before anthesis to carry 25 flowers on each of 5 branches. One group was treated with GA_3_ (30 ppm), and the control group was treated with water. The analysis was carried in cvs. Thompson Seedless (TS), Black Finger (BF), Spring Blush (SB), and Early Sweet (ES). All the other details are as described in Figs. 4 and 5.

To test if similar regulation occurs in other stenospermocarpic varieties, we analyzed the percent fruit set in cvs. Spring Blush, Thompson Seedless, Black Finger, and Early Sweet manipulated to carry 125 flowers (Fig. 6). Compared to their various controls, GA (30 ppm) significantly increased fruit set, which reached 70–80% in all four varieties. Interestingly, under the control treatment, the percent fruit set of cv. Early Sweet was more than twofold higher than the other cultivars.

**Fig. 7:**
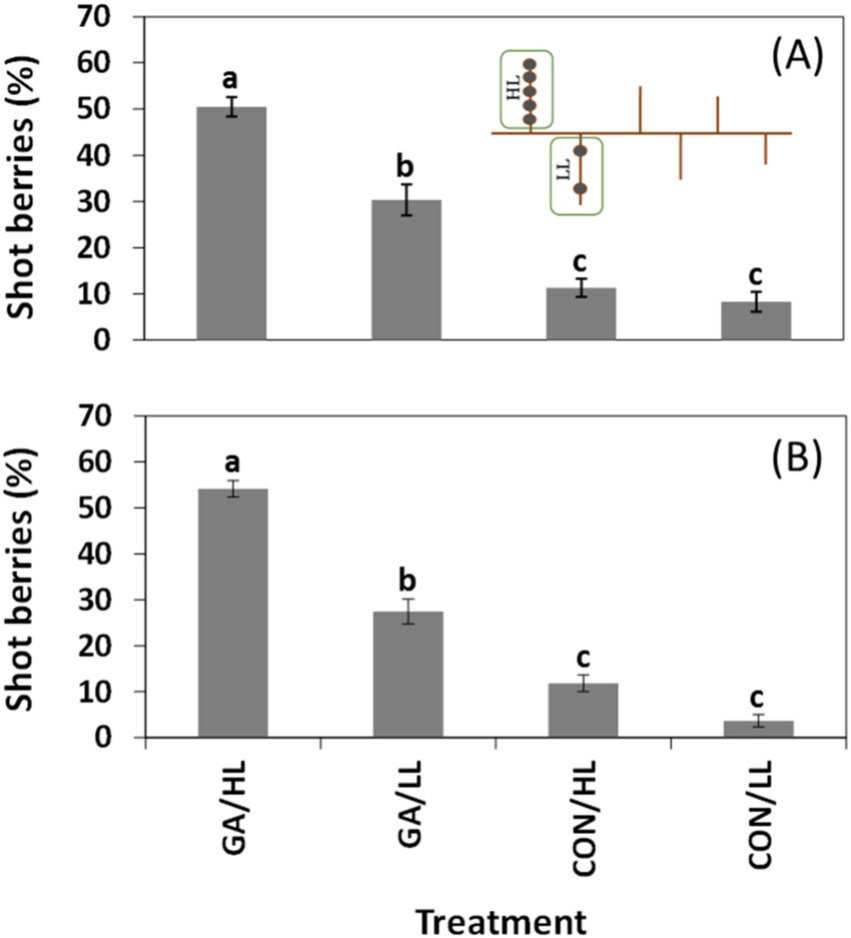
The effect of differential flower load on neighboring shoulders within a cluster on shot berry percentage. Two groups of 25 inflorescences were thinned to carry 10 flowers on one shoulder (low load, LL) and 40 flowers on the opposite shoulder (high load, HL). During the following season, two groups of 50 clusters were used, and HL shoulders carried 50 flowers. For additional details on the cluster, design see methods section. In both experiments, one group was treated with GA_3_ (30 ppm), and the other group was treated with water. At harvest, HL and LL shoulders were sampled and analyzed. The percentage of shot berries in HL and LL shoulders of GA-treated and control clusters in the two consecutive seasons is presented, labeled as (**a**) and (**b**). All the other details are as described in Figs. 1 and 4.

We also analyzed the effect of GA on shot berry formation on neighboring shoulders within the same inflorescence, carrying high and low loads of flowers (Fig. 7). Results from two consecutive growing seasons revealed (1) significant difference in the percentage of shot berry in branches with high and low flower load carried on GA-treated clusters; (2) small and non-significant difference between high- and low-load shoulders in untreated clusters; (3) increased percentage of shot berry in GA-treated clusters, compared with control.

**Fig. 8:**
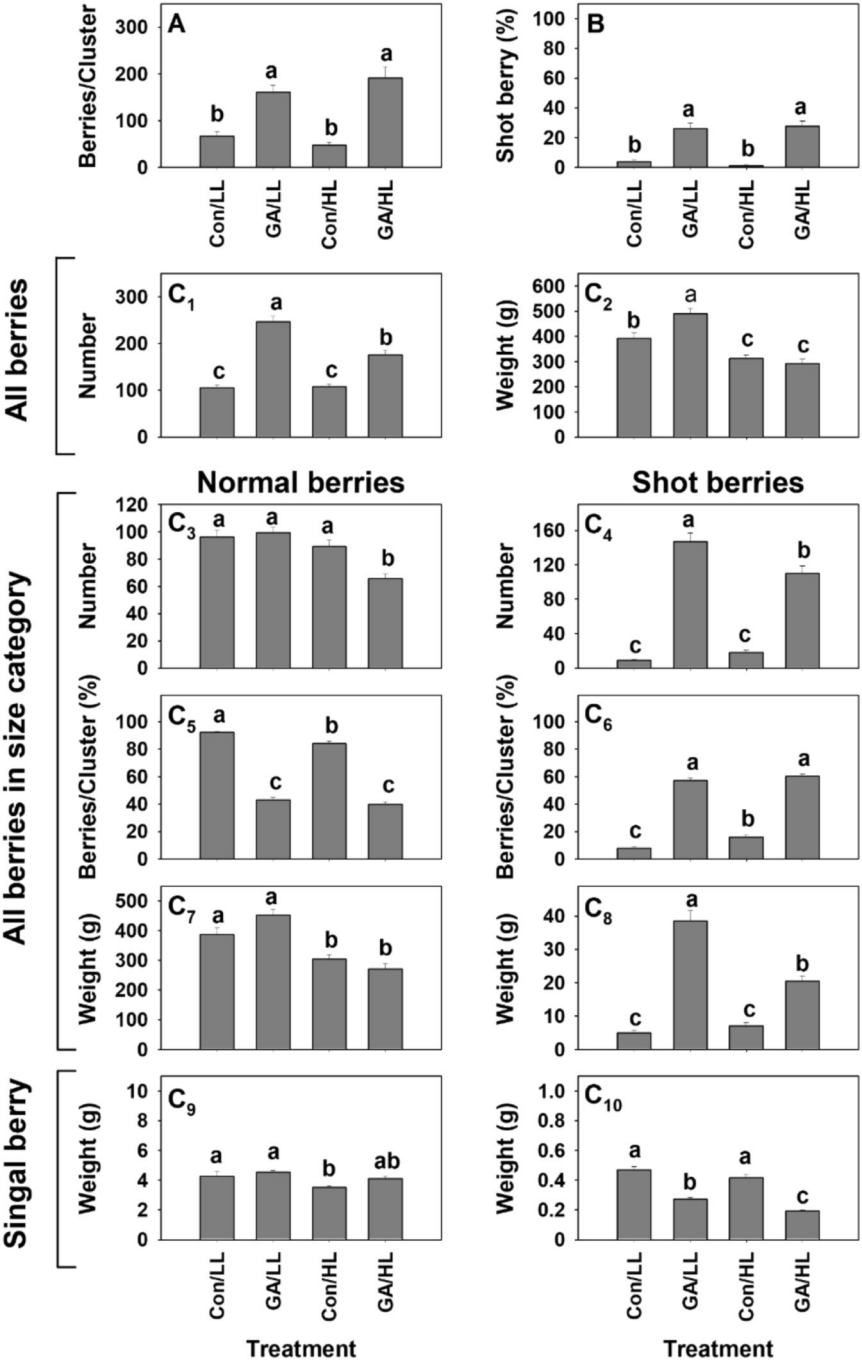
The effect of the initial number of inflorescences/vine on shot berry percentage in cv. Early Sweet clusters. Two groups of 10 vines were manipulated to carry 8 or 42 clusters on a vine, and termed LL and HL, respectively. On each vine, 4 clusters were randomly selected and treated. The selected clusters on half the vines in each group were treated with GA_3_ (30 ppm), and those on the rest of the vines in the group were treated with water. **a** Average number of berries per GA-treated and control clusters in LL and HL treatments that were carried out in the first season. **b** Percentage of shot berries per cluster in the treatments detailed in (**a**). **c**_1_–**c**_10_ Detailed analysis of numbers and weights of all the berries per cluster, berries per size category, and weight of single berry per size category in the experiment that was performed during the following season, using 24 vines per group. All the other details are as described in Figs. 1 and 4.

The effect that the number of inflorescences on a vine has on the formation of shot berries was also analyzed (Fig. 8). GA treatment resulted in a higher number of berries per cluster (Fig. 8a) and a larger fraction of small berries (Fig. 8b), compared to control. However, when comparing GA-treated clusters from vines carrying 8 (low load, LL) or 42 clusters (high load, HL), no significant difference was found for shot berry percentage.

To further study the effect of GA treatment under different cluster loads, an identical experiment of a larger scale was conducted (Fig. 8c). In agreement with the data described in Fig. 8a and b, GA treatment resulted in a higher number of berries per cluster (Fig. 8c_1_) and a higher percentage of shot berries in both LL and HL (Fig. 8c_6_), when compared to the relevant controls.

Detailed cluster analysis also revealed that (1) cluster load had no effect on the number of berries/cluster when control clusters were compared (Fig. 8c_1_). Similarly, load had no significant effect on the number of normal berries (Fig. 8c_3_), and the number of shot berries (Fig. 8c_4_) in control cluster. However, a significant increase in the percentage of small berries per cluster was recorded when the cluster load on the vine was increased (Fig. 8c_6_); (2) when GA-treated clusters are compared, the data indicate that HL induced significant decrease in number of berries/cluster, number of normal berries/cluster and number of shot berries/cluster (Fig. 8c_1_, 8c_3_, and 8c_4_, respectively). However, the proportion of normal berries/shot berries (Fig. 8c_6_) in GA-treated clusters was not affected by reduction in the number of berries in both categories; (3) when compared to control, GA did not affect the number of normal berries under LL, and significantly reduced it under HL (Fig. 8c_3_); (4) comparison of each treatment in HL and LL revealed that in both GA-treated and control clusters, increased cluster load resulted in a decrease of both cluster weight (Fig. 8c_2_), and weight of normal berry fraction (Fig. 8c_7_). The description above also applies to single normal berry weight (Fig. 8c_9_) in the control; (5) in control clusters, increased load has no significant effect on the weight of shot berry fraction (Fig. 8c_8_) and the weight of single shot berry (Fig. 8c_10_). When GA-treated clusters from both cluster loads are compared, HL results in significant decrease in weight of both shot berry fraction (Fig. 8c_8_) and single shot berry (Fig. 8c_10_); (6) when compared to control, GA had no significant effect on weight of normal berry fraction (Fig. 8c_7_) or single normal berry weight (Fig. 8c_9_) in both loads. On the other hand, GA treatment resulted in a considerable increase in shot berry fraction weight (Fig. 8c_8_) and a significant decrease in single shot berry weight (Fig. 8c_10_), in both loads, as indicated above.

## Discussion

Since true seedless cultivars (parthenocarpic cultivars) are rare in *Vitis vinifera*, the worldwide industry of ‘seedless’ table grape relies on stenospermocarpic cultivars. In these cultivars, where seeds serve as the primary source of GA only prior to abortion^25^, the berries are usually small due to relatively low levels of bioactive GA^13^. Therefore, stenospermocarpic varieties are treated with exogenous GA to stimulate berry development to a commercially acceptable size. Application of exogenous GA is also used for rachis stretching and cluster thinning, which allows the proper spacing between berries^15,16,18^. Yet, issues of timing of application and differential sensitivity of organs and cultivars frequently result in adverse effects due to the involvement of GA in the regulation of multiple aspects of plant development. For instance, GA application for sizing may reduce fruitfulness in succeeding season by promoting the development of uncommitted primordia to tendrils^26^, can delay ripening, and can lead to increased shatter due to enhanced rachis thickness^15,21,27^.

This study focuses on understanding the adverse effect of GA application on shot berry production in stenospermocarpic cultivars, a phenomenon often reported by growers and extension specialists and supported by some publications^1,21–24^. Cv. Early Sweet is very susceptible to shot berry formation, and thus provides a prism through which we tried to dissect the interaction between GA and shot berry formation.

A common assumption is that the cause for shot berry production is a failure of parthenocarpic fruitlets to respond to GA sizing treatment and develop to normal size berries, like their stenospermocarpic neighbors. Hence, we initially used cv. Early Sweet to (1) test the ability of GA to induce shot berry formation; (2) test the ability of GA to induce parthenocarpy; (3) follow the development of the parthenocarpic berries and test the assumption that they develop into shot berries.

Our data revealed that the application of GA to cv. Early Sweet clusters 2 weeks before anthesis or at anthesis, resulted in intense shot berry formation (Figs. 1 and 3). This intensity was found to be concentration-dependent, as it was significantly increased by using 30 ppm instead of 10 ppm GA_3_ (Fig. 3). Interestingly, the higher GA_3_ concentration resulted in a smaller size of shot berries (Fig. 1b) and a bigger size of the “Big” berries (data not shown), implying that GA had induced competition between berries from the two size categories. Our data also confirmed that the application of GA to emasculated flowers induces parthenocarpic fruit set in the cv. Early Sweet variety (Fig. 2).

In contrast to the initial assumption, parthenocarpic berries that developed from emasculated flowers had the capacity to develop into big berries. Moreover, the appearance of emasculated clusters was similar to that of non-emasculated clusters with the same initial flower load. Interestingly, emasculation without exogenous GA treatment led to inflorescence death, suggesting that GA supply from the anthers or the calyptra is indispensable for inflorescence survival until fruit set (Fig. 2b). In keeping with these findings, the application of a GA biosynthesis inhibitor had a similar negative effect on the survival of non-emasculated inflorescences (Fig. 2c). Stamens appear to be the main site of bioactive GA synthesis in flowers and the source for GA transport to nearby flower tissues^28^, and our data suggests that they may serve as the GA source for the rachis as well. The results also suggest that the early application of GA to non-emasculated flowers will result in parthenocarpic fruit set as well (Figs. 1 and 2). This insight was useful in our following experiments.

Our data clearly showed that inflorescences with decreased flower load presented significantly lower shot berry intensity when compared to that of inflorescences with a much higher number of flowers that were exposed to identical GA treatment (Fig. 3). These data suggest that while GA indeed induces parthenocarpic fruit set, it is not the parthenocarpic nature per se that destines the berry to become a shot berry. Instead, the initial flower load on an inflorescence may be the primary variable that regulates shot berry intensity. In support, the gradual increase of initial flower load resulted in a gradual increase in shot berry percentage within GA-treated clusters (Fig. 4). Separate statistical analyses exposed a similar trend in control inflorescences. These findings may imply that (1) flower load has a role in the regulation of shot berry intensity, regardless of GA application; (2) by forcing fruit set, GA is able to bypass such a mechanism, which possibly regulates earlier steps in the developmental cascade that lead to fruit set. The above corresponds well with the negative correlations between the flower load and the percentage of fruit set (Fig. 5) and with reduced fruit set variability in the presence of GA (Figs. 5 and 6). Interestingly, it appears that natural fruit set in cv. Early Sweet is significantly higher compared with the other varieties that we tested (Fig. 6). This limited ability to balance fruit set levels may be the source for increased size variability in cv. Early Sweet, which correlates with increased variability observed following GA-induced fruit set.

Cross-talk between flower load, fruit set and berry size have been formerly considered as part of the regulation of assimilates distribution between competing sinks in grapevine. Sucrose, which is produced in photosynthetically active tissues, is transported via the phloem vascular system to heterotrophic “sink” tissues that are dependent on imported sucrose for growth^29^. The import rate of an organ depends on its sink strength, relative to the strength of all other sinks on the plant, which compete for assimilates. The relative priority of a particular sink depends on its ability to store and metabolize sucrose, which reflects its assimilates demands, and determines its capability to maintain a favorable pressure gradient to the source. Therefore, sink strength is affected by the organ size and its activity, and modification of these parameters may modify assimilates transport. For example, grapevine inflorescence is a poor competitor until bloom, but fruit set induces a change in sink strength, which allows the cluster to dominate the hierarchy of sink priorities^30–35^.

Naturally, when greater number of sinks compete for a given source, less is available for each sink. In agreement, studies of relevant issues in grapevine revealed that (1) increasing number of berries per unit leaf area can limit berry expansion, (2) early loss of fruit, due to poor fruit set or early thinning, is partly compensated by increased berry size of the remaining fruits, (3) vines adjust their requirements by limiting the percentage of fruit set and the berry size, and (4) removal of sinks before bloom may result in increased percentage of fruit set and increased berry size^35^.

While the competition-related literature above reflects various aspects of our findings, the differential behavior of sinks of similar hierarchy within the cluster remains a puzzling phenomenon, in light of the fact that they are served by the same phloem strand. The assumption that individual berries differentially control delivery of phloem solutes was raised before, and maybe supported by the known fact that different berries on the same cluster accumulate different amounts of sugar. Below we integrate our data from several experiments and propose a model that addresses this issue, which is in line with a formerly proposed speculation that flowers with initial advantage prior to bloom remain more competitive throughout out their growth and ripening^35–38^.

Integration of the data from detailed cluster analyses in several experiments (Figs. 1, 4b, 8c and Fig. S1) suggests that when a high number of pre-anthesis flowers is available, GA induces (1) a significant and rather dramatic increase in shot berry percentage per cluster; (2) a significant increase in the weight and number of berries in the shot berry size category; (3) a significant decrease in the average size of a shot berry. Such effects were not observed where GA was applied to inflorescences with low flower load (Figs. 3 and 4). Data from these experiments also indicate that pre-anthesis application of GA to inflorescences with high load of flowers results in significant increase in the total number of berries per cluster, but this increase is not accompanied by significant increase in cluster weight. Interestingly, GA treatment did not lead to a significant increase in the number of berries in the normal berry fraction, or in its total weight.

While in our first experiment (in which categorization of sizes was based on visual impression) GA treatment resulted in increase in average weight of a big berry, it did not lead to a similar increase in the average weight of a normal berry in the following experiments (see Figs. 4b and 8c), in which analytical size classification was applied. In light of the known effect of GA on sizing, this result was initially surprising. Therefore, we followed the behavior of size sub-categories within the normal berry fraction (Fig. S2). The data indicates that under high flower load GA treatment results in (1) a significant decrease in the percent of big berries within the group of normal size berries; (2) a significant increase in the average big berry weight, without significant change in the total weight of all big berries; (3) a significant decrease in the weight of medium size berries.

The data described above suggest a zero-sum like scenario, in which each fraction gain or loss is balanced by the losses and gains of the other fractions. Limited availability of resources/cluster may dictate certain weight of a cluster, which is achieved by interplay between berry number and berry size. The main forces that affect this interplay are high number of flowers, initial variability in sink capacity of different flowers, and GA-induced fruit set and sizing.

The cluster load on the vine may have an effect, if limited source and competition between sinks is considered. Interestingly, (1) significant decrease in cluster load in LL treatment did not prevent shot berry development in clusters with high flower load when fruit set was increased by GA (Fig. 8c), and (2) shot berry percentage was similar in LL and HL treatment.

A unique situation was observed when GA-treated clusters from LL and HL treatments were compared. Pre-anthesis application of GA under a high load of clusters resulted in a lower number of both normal and shot berries. We speculate that GA-induced fruit set was limited under high cluster load, compared to GA-induced fruit set under low cluster load, resulting in a relative decrease in total berries per cluster. However, since the number of fruitlet/cluster was still high in GA-treated clusters under HL (about 180 berries), it resulted in similar percentage of shot berries.

In light of the above, we assume that competition between flowers within the cluster is more important than the competition between clusters for potential shot berry development. Moreover, the competition appears to be rather local, since branches with different flower loads present different intensity of shot berries (Fig. 7). An interesting open question is which flowers on a high-load branch are destined to develop into shot berries and what is the basis of such differential sensitivity.

Cluster load per se is not the focus of the current study, but it may be valuable to note that in control clusters, carrying about 100 berries in both cluster load treatments, HL resulted in a significant decrease in total weight, in weight of the normal berry fraction, and in the weight of single normal berry. There was also a significant increase in the shot berry percentage, but it was not accompanied by significant effects on the number of shot berries/cluster, shot berry fraction and weight of a single shot berry. This data suggest that cluster load is effecting cluster and berry size, as expected, but may have a less pronounced effect on shot berries development.

We propose the following hypothetical scenario to explain our findings. Inflorescence can be described as a system with a single source, and a population of sinks of a similar nature and a given initial variability. Following fruit set, the size of the sinks population is fixed. When the number of the sinks is reduced, the total available assimilates may be greater than or equal to the level required for optimal development of a single sink. In such a case, initial variation between the sinks will not be reflected, and similar development of all sinks will commence. Accordingly, an increase in the number of sinks will naturally reduce the resources available for the development of the single sink. Once a certain threshold is exceeded, the initial variability among sinks will be expressed and result in the differential ability to import assimilates within the population. Moreover, the development of fruitlets with better initial ability to import assimilates will be faster, and lead to further escalation of competition due to further increase in their sink strength.

Exposure to GA may affect the scenario on two levels. Primarily, GA will increase the final number of fruitlet sinks developed from a given flower population by increasing the percentage of fruit set. Additionally, the demonstrated ability of GA to encourage translocation of assimilates to fruitlets^39^ may exacerbate competition among fruitlets with an initial difference in assimilates import abilities. The decreased weight of shot berries in response to increased GA concentration (Fig. 1b), despite the similarity in shot berry fraction size, supports such a scenario. Understanding the nature of the variability within the flower population that drive differential development of fruitlets under high-load conditions is an exciting future challenge.

## Materials and methods

### Plant material

Most of the experiments were carried out with *V. vinifera* cv. Early Sweet. Field experiments were carried out using mature vines (8–10 years old) grafted on Richter, located at a commercial vineyard in Petahya, Judea plains, and Israel (32°52′09.5″N and 34°53′16.1″E). The vines were spaced 3 m within rows and 1.5 m between rows, grown on heavy soil with drip irrigation, trained on a Y-shaped trellis on a bilateral cordon. The vineyard was subjected to all the standard cultural practices. Other experiments were conducted with cvs. Thompson Seedless, Black Finger, and Spring Blush that are grown in vineyards located at Petacya, Lachish, and Avigdor, respectively, and similar details apply.

### Chemicals

“ProGibb” (33 g GA_3_/L; Valent Biosciences, CA, USA) was used for GA_3_ treatments. “Cultar 25” (250 g paclobutrazol/L; Syngenta, Basel, Switzerland) was used for Paclobutrazol (PAC) treatments. All the solutions, including water control, contained Triton X-100 (0.025%) (Sigma-Aldrich, St. Louis, MO, USA), which was used as a surfactant.

### Induced shot berry formation by application of GA to inflorescences

In nine groups of 10 inflorescences, all but 5 uppermost branches were removed. Four out of the nine groups were each dipped in GA_3_ (10 ppm, 30 ppm), paclobutrazol (250 ppm), or water.

In the remaining five groups, all five branches were thinned to carry 10 flowers each, producing inflorescences with 50 flowers. In two of these 5 groups, the flowers were emasculated by manual removal of calyptra and anthers. The other three groups were kept non-emasculated. Pairs of emasculated and non-emasculated groups were dipped in GA_3_ (10 ppm) or water. The last group of non-emasculated 50 flowers, containing inflorescences, was treated with a higher concentration of GA_3_ (30 ppm). The inflorescences were bagged to prevent cross-pollination. Bags were removed only after fruit set. The experiment was repeated at two developmental stages: 2 weeks before anthesis (BA) and at anthesis (A).

### Manipulation of flower load on inflorescence

Six groups of 5 inflorescences were thinned to carry 5 uppermost branches. Two groups of inflorescence were thinned to carry 155 flowers as follows: 40 flowers on each shoulder, and 25 flowers on the 3 branches below. Two groups were thinned to carry 100 flowers consisting of 20 flowers on each branch, while another two groups carried 50 flowers (10 flowers on each branch). One group from each cluster design was treated with GA_3_ (30 ppm), and the other treated with water as a control. The experiment was repeated in the succeeding growing season using six groups of 25 inflorescences.

### Manipulation of flower load on neighboring shoulders

In the first season, 2 groups of 25 inflorescences were thinned to carry 10 flowers on the left shoulder and 40 flowers on the right shoulder. In the two branches immediately below, the orientation of flower load adjustment was reversed, while the fifth branch was thinned to carry 25 flowers. In the following season, 2 groups of 50 clusters were thinned to carry 7 branches. The two shoulders of each inflorescence were thinned to carry 10 and 50 flowers. In the 5 branches below, the flower number was not modified. At harvest, only shoulders were sampled and analyzed.

### Manipulation of inflorescence load on the vine

Two groups of 10 vines in the first season and 24 vines in the following season were manipulated to carry 8 (LL) or 42 (HL) clusters on a vine. On each vine, 4 clusters were randomly selected and treated. Half the vines in each group were treated with GA_3_ (30 ppm) and the other half with water, as a control.

### Analysis of fruit set percentage

To test the effect of GA on fruit set percentage, 2 groups of 25 inflorescences were thinned 2 weeks prior to anthesis, to carry 25 flowers on each of 5 branches. One group was treated with GA_3_ (30 ppm), and the other treated with water. The analysis was carried in cvs. Thompson Seedless, Black Finger, Spring Blush, and Early Sweet. Berry number/cluster was determined as detailed in “Data Collection and analysis”, and used to calculate fruit set percentage using initial flowers number as 100%.

### Data collection and analysis

At harvest, clusters were collected and brought to the lab. Data were collected for each cluster separately. All the berries were detached and subjected to size segregation. Initially, berries were visually assessed and categorized into “big” or “small” pools. This procedure resulted in size variation within a size category between GA-treated and control clusters (see Fig. 1b). Thus, in the next growing seasons, berries were subjected to size segregation using three size categories, i.e., big (B; >13 mm); medium (M; 7–12.99 mm); and small (S; <7 mm). The number of berries was recorded for each size category. Berries from the S category were considered shot berries, and berries from the B and M categories were considered normal berries. To calculate the total number of berries in each individual cluster, the number of berries in each size group was summed (total = big + medium + small). The percentage of shot berries in the relevant cluster was calculated. The representative fraction of shot berries per treatment was calculated by averaging the S fraction value for each cluster subjected to the respective treatment. Means and standard errors were calculated using data from all clusters per treatment. Statistical analysis was carried using the Tukey HSD test (*p* < 0.05). Similar procedures of data collection and processing were adopted for all the other parameters that are presented, including number and weight of all the berries/cluster, number and weight of all the berries in the normal and shot berry size categories, average weight of a single normal berry and shot berry.

## Supplementary information

Supplemental Figures
