# Supplementary material for: Gibberellin induced shot berry formation in cv. Early Sweet is a direct consequence of high fruit set
Source: Hortic Res. 2020 Oct 1;7:169. doi: 10.1038/s41438-020-00388-9 (PMC7528092; doi:10.1038/s41438-020-00388-9)
Supplement: Supplementary file 1 — Supplemental Figures [file 41438_2020_388_MOESM1_ESM.docx]

**SUPPLEMENTAL FIGURES**

**
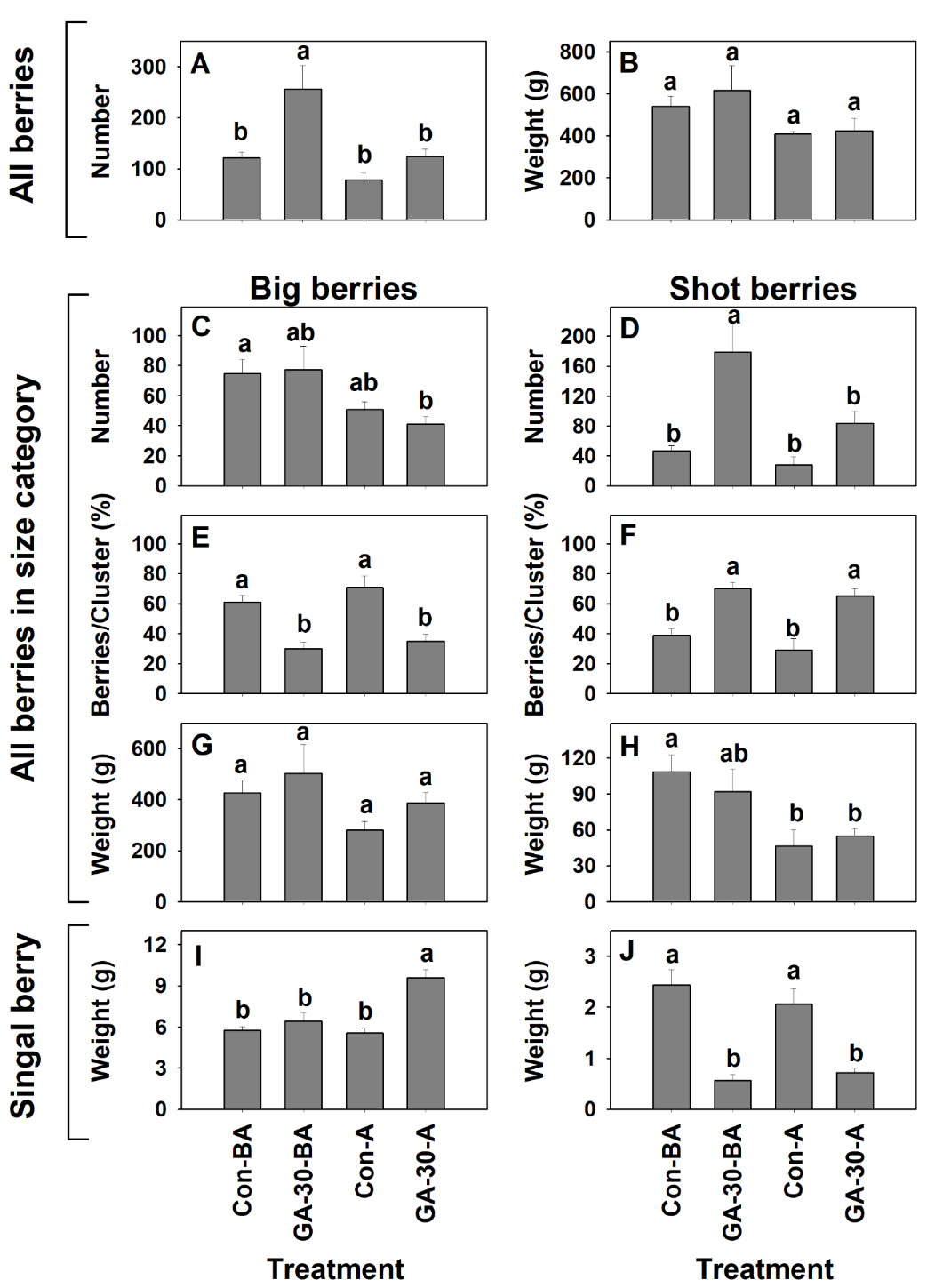
**

**Figure S1: Detailed analysis of the effect of GA application on various cluster size parameters.** Numbers and weights of all berries per cluster, berries per size category, and weight of single berry per size category in the experiment that is described in Figure 1 are presented. All the other details are as described in Fig. 1 and 4.


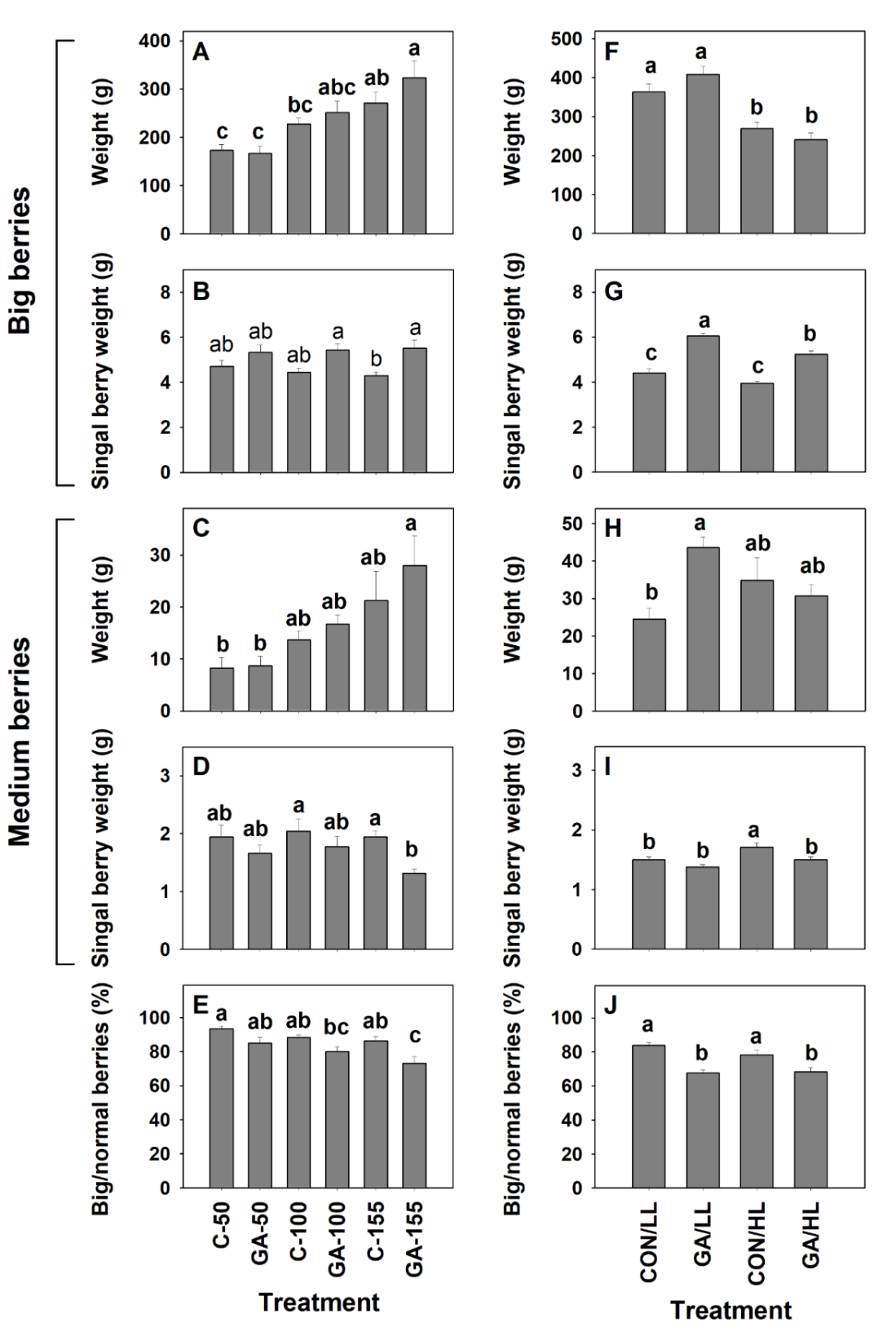


**Figure S2: The effect of gradual manipulation of initial flower load, and initial number of inflorescences on a vine,** **on the numbers and weights of berries in Big and Medium sub-categories within the normal berry fraction.** The data are related to the experiments that are described in Figure 4B and Figure 8C. All the other details are as described in Fig. 4 and 8, respectively.
